# Lessons Learned from Whole Exome Sequencing in Multiplex Families Affected by a Complex Genetic Disorder, Intracranial Aneurysm

**DOI:** 10.1371/journal.pone.0121104

**Published:** 2015-03-24

**Authors:** Janice L. Farlow, Hai Lin, Laura Sauerbeck, Dongbing Lai, Daniel L. Koller, Elizabeth Pugh, Kurt Hetrick, Hua Ling, Rachel Kleinloog, Pieter van der Vlies, Patrick Deelen, Morris A. Swertz, Bon H. Verweij, Luca Regli, Gabriel J. E. Rinkel, Ynte M. Ruigrok, Kimberly Doheny, Yunlong Liu, Joseph Broderick, Tatiana Foroud, FIA Study Investigators

**Affiliations:** 1 Department of Medical and Molecular Genetics, Indiana University School of Medicine, Indianapolis, Indiana, United States of America; 2 Department of Neurology and Rehabilitation Medicine, University of Cincinnati School of Medicine, Cincinnati, Ohio, United States of America; 3 Center for Inherited Disease Research, Johns Hopkins University; Baltimore, Maryland, United States of America; 4 Department of Neurology and Neurosurgery, Brain Center Rudolf Magnus, University Medical Center Utrecht, Utrecht, the Netherlands; 5 Department of Genetics, University Medical Center Groningen, University of Groningen, Groningen, the Netherlands; 6 Genomics Coordination Center, University Medical Center Groningen, University of Groningen, Groningen, the Netherlands; 7 Department of Neurosurgery, University Hospital Zurich, Zurich, Switzerland; University of North Carolina, UNITED STATES

## Abstract

Genetic risk factors for intracranial aneurysm (IA) are not yet fully understood. Genomewide association studies have been successful at identifying common variants; however, the role of rare variation in IA susceptibility has not been fully explored. In this study, we report the use of whole exome sequencing (WES) in seven densely-affected families (45 individuals) recruited as part of the Familial Intracranial Aneurysm study. WES variants were prioritized by functional prediction, frequency, predicted pathogenicity, and segregation within families. Using these criteria, 68 variants in 68 genes were prioritized across the seven families. Of the genes that were expressed in IA tissue, one gene (*TMEM132B*) was differentially expressed in aneurysmal samples (n=44) as compared to control samples (n=16) (false discovery rate adjusted p-value=0.023). We demonstrate that sequencing of densely affected families permits exploration of the role of rare variants in a relatively common disease such as IA, although there are important study design considerations for applying sequencing to complex disorders. In this study, we explore methods of WES variant prioritization, including the incorporation of unaffected individuals, multipoint linkage analysis, biological pathway information, and transcriptome profiling. Further studies are needed to validate and characterize the set of variants and genes identified in this study.

## Introduction

Subarachnoid hemorrhage (SAH) is the most devastating subtype of stroke. Fatality from SAH between 21 days to one month of the hemorrhage ranges from 25–35% in high-income countries to almost 50% in low- to middle-income countries [1]. Up to 80–90% of SAH cases are caused by rupture of intracranial aneurysms (IA), which are present in approximately 3% of the population [2]. Smoking and hypertension are important risk factors, increasing the risk of IA rupture by 3.1 and 2.6 times respectively [3]. The risk of an IA and for IA rupture is also increased among individuals having a first-degree relative with a history of an IA [2, 4, 5]. The location and number of IAs in a given individual also appears to be influenced by a family history [6]. Thus, several lines of evidence suggest that IA is due to both genetic and environmental risk factors. Unfortunately, until more is understood about these risk factors, the severe morbidity and mortality associated with this disease will continue to be a large public health burden.

Several approaches have been employed to identify genes contributing to IA. Initial studies utilized pedigrees having multiple affected members. Analyses in these initial studies detected linkage to several chromosomal regions (1p34.3–36.13 [7, 8], 4q32.2 [9], 6p23 [10],7q11 [7], 7q36.3 [9], 8q12.1 [9], 11q24–25 [10–12], 12q21.33 [9], and 14q23–31 [12]); however, the causative gene was not identified in any of these regions. More recently, genomewide association studies (GWAS) have focused on the role of common variants that might individually have a small effect on disease risk. Analyses have consistently detected association to single nucleotide polymorphisms in *CDKN2BAS*, also known as *ANRIL*, on chromosome 9p21.3 [13–15], as well as *SOX17* on chromosome 8q12.1 [13–15]. Association has also been reported to *EDNRA* on chromosome 4q31 [16], *CNNM2* on chromosome 10q24 [14], *KL/STARD13* on chromosome 13q13 [14], and *RBBP8* on chromosome 18q11 [14]. Together, these genes only explain a fraction of the population attributable risk for IA.

Advances in technology, especially in the development of high-throughput sequencing, now make it possible to efficiently search for rare variants having a large effect on disease risk. These rare variants may point to novel genes and pathways that are critical to improve the molecular understanding of IA and methods of predicting those at greatest risk. In the present work, whole exome sequencing (WES) was applied to a unique set of families densely affected with IA to investigate the role of rare genetic variation in disease susceptibility and to demonstrate important study design considerations for WES studies in complex disease.

## Materials and Methods

### Families Selected for Whole Exome Sequencing

Individuals were recruited as part of the Familial Intracranial Aneurysm (FIA) Study [17]. Study approval was granted by institutional review boards at Indiana University, University of Cincinnati, Mayo Clinic, University of Medicine and Dentistry of New Jersey, Cleveland Clinic, Columbia University, University of Texas Houston, Indianapolis Neurosurgical Group, Goodman Campbell Brain and Spine, University of Western Ontario, University of Maryland, McGill University, University of Montreal, Notre Dame Hospital, Ackland UniServices, The George Institute, Royal Perth Hospital, Sir Charles Gairdner Hospital, Royal Adlaide Hospital, Royal Melbourne Hospital, Alfred Hospital, Royal North Shore Hospital, Westmead Hospital, Royal Prince Alfred Hospital, Northwestern University, University of Ottawa, University of Pittsburgh, Stanford University, University of California San Francisco, University of Southern California, University of Virginia, University of Washington, University of Manitoba, University of Alabama Birmingham, Allegheny General Hospital, Brigham and Women’s Hospital, Massachusetts General Hospital, University of Florida, Johns Hopkins University, University of Michigan, and Washington University in St. Louis. Written consent was obtained from all study participants.

Families were recruited to ensure that DNA could be obtained from at least two living affected relatives and that the family would be informative for linkage analysis. Exclusion criteria included (i) a fusiform-shaped unruptured IA of a major intracranial trunk artery; (ii) an IA that is part of an arteriovenous malformation; (iii) a family or personal history of polycystic kidney disease, Ehlers Danlos syndrome, Marfan’s syndrome, fibromuscular dysplasia, or Moya-Moya disease; or (iv) failure to obtain informed consent from the patient or family members. To identify unruptured IA, magnetic resonance angiography (MRA) was offered to first degree relatives of affected family members who had a higher risk of IA as defined by: 1) 30 years of age or older and 2) either a 10 pack year history of smoking or an average blood pressure of ≥140 mmHg systolic or ≥90 mmHg diastolic.

Only individuals having an IA based on an intra-arterial angiogram, operative report, autopsy, or size ≥7 mm on non-invasive imaging (MRA) were considered “definite” cases (Table 1). Two neurologists independently reviewed each record to determine if a subject met all inclusion and exclusion criteria. In case of disagreement, a third neurologist reviewed the data.

**Table 1:** Disease phenotypes.

Classification	Definition
Definite	Medical records document an intracranial aneurysm (IA) on angiogram, operative report, autopsy, or a non-invasive imaging report (MRA, CTA) demonstrates an IA measuring 7mm or greater.
Probable	Death certificate mentions probable IA without supporting documentation or autopsy. Death certificate mentions subarachnoid hemorrhage (SAH) without mention of IA and a phone screen is consistent with ruptured IA (severe headache or altered level of consciousness) rapidly leading to death. An MRA documents an IA that is less than 7 mm but greater than 3 mm.
Possible	Non-invasive imaging report documents an aneurysm measuring between 2 and 3 mm or SAH was noted on death certificate, without any supporting documentation, autopsy or recording of headache or altered level of consciousness on phone screen. Death certificate lists ‘aneurysm’ without specifying cerebral location or accompanying SAH.
Not a case	There is no supporting information for a possible IA.

**Fig 1:**
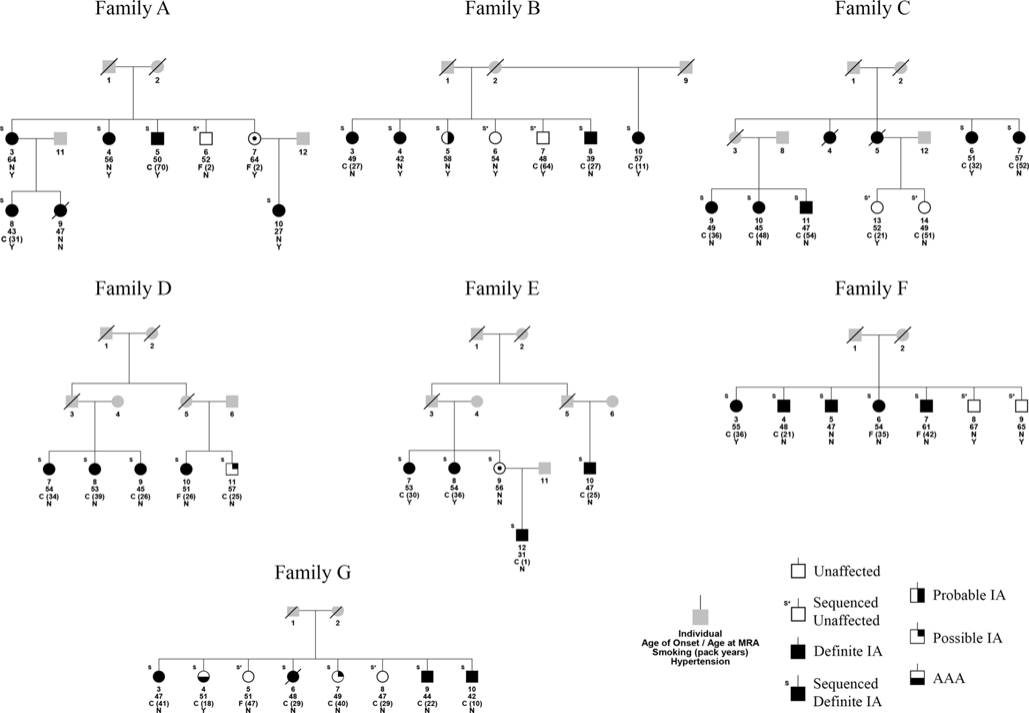
Simplified pedigrees for the 7 whole exome sequencing families. Only sequenced individuals and those needed to preserve generational structure are shown to protect the anonymity of the pedigree. IA = intracranial aneurysm. All affected individuals are definite IA unless noted as a probable IA, possible IA, or aortic abdominal aneurysm (AAA). Criteria for defining definite, probable, and possible IA statuses are outlined in Table 1. All unaffected individuals, with the exception of individual E-9, had an MRA performed that did not show evidence of an IA. Grey indicates an unknown phenotype. An ‘S’ above an individual denotes that the individual was selected for sequencing.

Seven families of European American descent with the highest density of affected individuals who also had DNA available were selected for WES [18] (Fig. 1). All affected individuals for which sufficient DNA was available were selected for sequencing. Unaffected individuals were selected only if there was an MRA conducted that confirmed the absence of an IA at 45 years or older and if there was sufficient DNA available. One clinically unaffected individual in family E was assumed to be an obligate carrier and was sequenced with her offspring to allow confirmation of allele transmission. Within the seven families, 45 individuals were chosen for WES.

### Whole Exome Sequencing

WES was performed at the Center for Inherited Disease Research (CIDR, Johns Hopkins University). Exonic sequences were captured using the Agilent SureSelect Human All Exon 50Mb kit, and paired-end sequencing was performed on the Illumina HiSeq 2000 system, using Flowcell version 3 and TruSeq Cluster Kit version 3. All samples were genotyped using the Illumina HumanOmniExpress-12v1_C platform for quality assurance. Two HapMap samples and two study duplicates were used to ensure library preparation batch quality.

### Whole Exome Sequencing Bioinformatics

Primary analysis was done using HiSeq Controls Software and Runtime Analysis Software. The CIDRSeqSuite pipeline was used for secondary bioinformatics analysis, which consists mainly of alignment using Burrows Wheeler Aligner (BWA version 0.5.9) [19] to the human genome reference sequence (build hg19) and applying the Genome Analysis Toolkit (GATK version 1.0.4705) [20] to perform local realignment and base quality score recalibration. Duplicate molecules were flagged and mate-pair information synchronized using Picard (version 1.52, http://picard.sourceforge.net/). The GATK Unified Genotyper (GATK version 1.2–29) was used for multi-sample variant calling. The dataset, consisting of called variants, subject phenotypes, and pedigree information for the multiplex IA families can be requested directly from the National Center for Biotechnology Information (NCBI) Database of Genotypes and Phenotypes (dbGaP) (http://www.ncbi.nlm.nih.gov/projects/gap/cgi-bin/study.cgi?study_id=phs000636.v1.p1) (accession phs000636). Mapped reads are available on the Sequence Read Archive (http://www.ncbi.nlm.nih.gov/sra) (accession SRX329208-SRX329252).

GATK Variant Quality Score Recalibration (VQSR, GATK version 1.2–38) [21] created a high-quality call set for SNVs by using an adaptive error model to estimate the likelihood of true genotype calls based on aggregating information across multiple quality metrics. As recommended by GATK, HapMap 3.3 and the Illumina Omni 2.5M chip sites, available from the GATK bundle 1.2, were used as training sets and the annotations of Quality by Depth, Haplotype Score, Mapping Quality Rank Sum, Read Position Rank Sum, Fisher Strand Bias Test, and Mapping Quality were used as quality metrics for the recalibration. SNVs were filtered until 99% of the overlapping HapMap 3.3 sites were retained after application of VQSR. Insertion/deletions were removed if they had a quality by depth < 2.0, ReadPosRankSum < -20.0 (Z-score from Wilcoxon rank sum test of alternative versus reference read position bias), Fisher’s Strand Bias > 200.0 (phred-scaled p-value using Fisher’s exact test to detect strand bias), and/or a homopolymer run > 5.

ANNOVAR [22] was used to annotate variants for location, predicted effect on the protein across three gene databases (RefSeq, UCSC, and Ensembl), and corresponding gene and transcript length. Allele frequencies within European American populations in 1000 Genomes (February 2012 release, http://www.1000genomes.org) [23] and the Exome Sequencing Project (ESP) (ESP6500 release with insertion/deletions and chromosome X and Y calls, http://evs.gs.washington.edu/EVS/) [24] were recorded using custom scripts. The scripts mapped variants to 1000 Genomes and ESP based on chromosomal position and reference and alternate alleles to determine allele frequencies. If a variant was not found in 1000 Genomes or ESP, the alternate allele frequency was set to 0. If a variant was found in both 1000 Genomes and ESP, the smaller alternate allele frequency was taken as the consensus frequency.

Variants were annotated for binned minor allele frequencies from 290 samples without a known cardiovascular phenotype that were exome sequenced at CIDR using identical capturing and sequencing technology, although SAMtools [25] was used for variant calling instead of GATK Unified Genotyper. Variants that were monomorphic across all samples were also flagged.

Variants were also annotated using custom scripts for Gene Ontology (GO) (http://www.geneontology.org) [26] terms that were hypothesized to play a role in IA pathophysiology. GO terms used included GO:0001944 (vasculature development), GO:0001570 (vasculogenesis), GO:0003018 (vascular process in circulatory system), GO:0005581 (collagen), GO:0005604 (basement membrane), and GO:0051541 (elastin metabolic process).

Two programs were used to predict the pathogenicity of SNVs: SIFT [27] and PolyPhen-2 [28]. Scores of damaging for SIFT, or possibly or probably damaging for PolyPhen-2, were accepted as evidence for pathogenicity. Two additional programs were used to analyze the effect of insertions and deletions: SIFT-INDEL [29] for those that cause a frameshift, and DDIG-in for those that do not cause a frameshift [30]. Variants were also annotated for C-scores from the Combined Annotation Dependent Depletion (CADD) webserver (http://cadd.gs.washington.edu) [31]. C-scores of 10 or greater, corresponding to the 10% most deleterious substitutions in the human genome according to CADD, were considered damaging predictions.

Biological filtering retained loci if they: 1) were autosomal variants; 2) were predicted to be nonsynonymous SNVs or insertion/deletions in an exonic and/or splicing region (within 2 bp of a splicing junction, as annotated by ANNOVAR) based on RefSeq, UCSC, and Ensembl annotations; 3) had an allele frequency in European American populations <1% (1000 Genomes, ESP); 4) had an allele frequency less than 1% in CIDR binned minor allele frequencies and were not monomorphic across all samples; 5) were predicted most likely to be damaging by CADD and by at least one other protein prediction program; and 6) segregated with all individuals with a definite IA and obligate carriers in at least one family. All alignments for variants passing these biological filters were visually inspected using the Integrated Genomics Viewer [32] to confirm presence of a variant. Visual inspection for each variant included reviewing read pair orientations, mappability, and soft-clipping; variants that were called nearby; overall depth of sequencing and genomic features that might have inhibited coverage at that locus; and repetitive sequence that might have influenced the position or variant allele called for the locus. In addition to the filters described above, insertion/deletions were also compared against a different dataset consisting of approximately 500 samples without a known cardiovascular phenotype. This comparison dataset used GATK Unified Genotyper (version 2.3–9) for variant calling and the Agilent SureSelect Human All Exon 51Mb capture kit. If the allele designations and/or positions did not match between the two datasets but were within 10 bp, manual review of both the IA and comparison BAM files with IGV was done to reconcile differences in allele designations and position assignments between the two datasets.

Loci were also annotated if they: A) segregated with all aneurysms (including probable and possible IA and the one abdominal aortic aneurysm case in family G) and B) were not found in any sequenced unaffected individuals, excluding assumed obligate carriers.

### Linkage

The 7 families were included as part of a larger linkage study of 2,317 individuals from 394 families using the 6K Illumina array [9]. Multipoint parametric linkage analysis (autosomal dominant inheritance, 1% disease allele frequency was performed using Merlin [33]. Only genotypic data from family members with definite IA and obligate carriers were included in the linkage analysis. WES variants were annotated for the highest LOD score obtained by linkage markers within a 10Mb window centered on the sequencing variant. A maximum possible LOD score for each family was calculated by simulating a hypothetical fully informative marker using the aforementioned model parameters and the pedigrees for each family.

### Tissue Collection for RNA Expression

Aneurysm biopsies from the aneurysm fundus distal to the clip were collected from patients undergoing neurosurgical clipping of an IA at the Department of Neurology and Neurosurgery in the University Medical Center Utrecht in the Netherlands. These patients were completely independent of the families included for WES. Patients undergoing surgery because of intractable epilepsy were included as controls, and part of a superficial cortical artery in the resected part of the brain was excised as control vessel tissue. Samples were collected from 44 aneurysm biopsies (22 ruptured, 21 unruptured, 1 with unknown rupture status) and 16 control biopsies. All samples were immediately snap frozen in liquid nitrogen less than 1 minute after excision and stored at -80°C until further use.

### RNA Isolation, Sample Preparation, and Sequencing

RNA isolation, sample preparation, and sequencing was conducted at the University Medical Center Groningen in Groningen, the Netherlands. Each sample was homogenized with zirconia/silica beads in the BeadBeater machine (BioSpec products, Inc.). After homogenization, total RNA was extracted and purified using an RNeasy microkit (Qiagen, Valencia, CA, USA) according to the manufacturer’s instructions. An initial quality check of the samples by capillary electrophoresis and RNA quantification for each sample was performed using the LabChip GX (PerkinElmer, Waltham, Massachusetts, USA). Samples with a minimum amount of 7 ng non-degraded RNA were selected for subsequent sequencing analysis. Sequence libraries were generated using the TruSeq RNA sample preparation kit from Illumina (San Diego, USA) using the Sciclone NGS Liquid Handler (Perkin Elmer). To remove contamination of adapter-duplexes, an extra purification of the libraries was performed with the automated agarose gel separation system Labchip XT (Perkin Elmer). The obtained cDNA fragment libraries were sequenced on an Illumina HiSeq2000 using default parameters (single read 1x100bp) in pools of 10 or 11 samples. Processing of the raw data, including a demultiplexing step, was performed using Casava software (Illumina) with standard settings.

### Differential Expression Analysis

Sequencing reads with quality score under Phred Score <30 were discarded. The quality filtered trimmed fastQ files were then aligned to the human reference genome (hg19) using the STAR aligner [34], allowing for 2 mismatches. SAMtools version 0.1.18 [25] was used to sort the aligned reads. Gene level quantification was performed by HTSeq-0.5.4 [35] using parameters—mode = union—stranded = no and Ensembl version 71 as the gene annotation database.

R version 3.1.0 was used for differential expression analysis. The counts per gene for each sample obtained after alignment were used as input for the analysis. Low count genes (genes with less than 1 read per million in *n* of the samples, where *n* is the size of the smallest group of replicates, i.e. *n* = 16) were filtered out since there is little power to detect significant evidence of differential expression in these genes [36].

The Bioconductor (version 2.14) packages edgeR (version 3.6.2) and limma (version 3.20.2) were used for subsequent steps. To correct for technical influences, edgeR adjusts for varying sequencing depths between samples and normalizes for the RNA composition of the sample. A generalized linear model was used to test for differential expression between aneurysmal and control tissue. Other factors included in the model were age and sex of patients, as well as rupture status. Common and tagwise dispersion estimates were calculated with the Cox-Reid profile adjusted likelihood method to be able to correct for the technical and biological variation when fitting the multivariate negative binomial model. In estimating the tagwise dispersion, the program default for degrees of freedom (df = 10) was used. A negative binomial generalized log-linear model, using the tagwise dispersion estimates, was fitted to the read counts for each gene, and a gene-wise statistical test was performed. Then, a likelihood ratio test was performed. Benjamini Hochberg false discovery rates (FDR) for a transcriptome-wide experiment were calculated to correct for multiple testing. All genes with an FDR adjusted p-value <0.05 were considered individual genes of interest.

## Results

### Sequencing Data Quality

The average study duplicate reproducibility of SNV and insertion/deletion calls were 99.13% and 94.42%, respectively, and genotypes for non-reference calls per sample from the WES data achieved an average 99.57% concordance with genotype calls from the Illumina HumanOmniExpress-12v1_C array. The average sensitivity to heterozygote calls on the array was 98.13%. After application of GATK quality filters, 98,351 SNVs and 5,851 insertion/deletions were retained. The transition-transversion ratio for exonic variants and percent of SNVs in dbSNP 137, both measures of the quality of the data, were 3.3 and 94.79% respectively.

### Biological Filtering

The number of variants retained after each biological filter employed in the Methods is shown in Tables 2–3 for SNVs and insertion/deletions, respectively. The list of SNVs and insertion/deletions satisfying biological filters 1–6 is shown in Table 4. The final candidate variants passing biological filters 1–6 and manual inspection include 67 SNVs and 1 deletion. The sets of variants that A) segregate with all aneurysmal phenotypes and B) are not carried in unaffected individuals are included in Table 4 as subsets of the 68 final variants. The limitations of only considering these sets of variants are described in the Discussion.

**Table 2:** Single nucleotide variant filtering pipeline.

Family	A	B	C	D	E	F	G	All
All variants found in at least one definite IA	46168	41978	44689	44515	49142	39495	37809	98351
(1) Autosomal variants	45390	41280	43994	43701	48376	38925	37251	96552
(2) Variants predicted to be functional	12261	11158	11849	11841	13203	10578	10025	29194
(3) Rare variants	1020	889	953	1298	1356	843	823	7845
(4) Variants not found or of low frequency in the internal allele frequency database	793	725	740	1028	1049	676	658	6428
(5) Variants predicted damaging	393	345	369	442	470	297	306	3008
(6) Variants segregating with all definite IA in at least one family	13	11	2	10	4	8	24	67
Variants passing visual inspection	13	11	2	10	4	8	24	67
A. Variants segregating with all IA (definite, probable, possible) or AAA in at least one family	13	9	2	8	3	8	7	46
B. Variants not found in unaffected individuals	5	2	1	7	3	1	0	19

Numbers in parentheses refer to filtering steps described in the Methods. IA = intracranial aneurysm

**Table 3:** Insertion deletion filtering pipeline.

Family	A	B	C	D	E	F	G	All
All variants found in at least one definite IA	3316	2736	3226	3166	3396	2987	2966	5851
(1) Autosomal variants	3264	2705	3178	3102	3345	2940	2921	5737
(2) Variants predicted to be functional	538	457	560	541	581	511	465	1126
(3) Rare variants	284	221	299	277	299	266	260	589
(4) Variants not found or of low frequency in the internal allele frequency database	178	159	188	171	192	165	157	453
(5) Variants predicted damaging	60	59	65	50	59	55	42	194
(6) Variants segregating with all definite IA in at least one family	24	22	23	19	23	24	19	26
Variants passing visual inspection and manual review with internal database calls	0	0	0	0	0	0	1	1
A. Variants segregating with all IA (definite, probable, possible) or AAA in at least one family	0	0	0	0	0	0	0	0
B. Variants not found in unaffected individuals	0	0	0	0	0	0	0	0

Numbers in parentheses refer to filtering steps described in the Methods. IA = intracranial aneurysm

**Table 4:** Candidate variants identified through whole exome sequencing in 7 multiplex families.

Chr	Position	Ref	Alt	Gene	Full_Name	Alt Freq	PolyPhen	SIFT	CADD Cscore	Amino Acid Change	LOD	Family	Unaff	logFC	FDR
1	6631121	C	T	TAS1R1	taste receptor, type 1, member 1	0.0001		+	16.77	NM_177540:exon2:c.C344T:p.T115M	1.08	D§	0	N/A	N/A
1	15905363	G	T	AGMAT	agmatine ureohydrolase (agmatinase)	0.0026		+	15.62	NM_024758:exon4:c.C711A:p.N237K	0.83	F	1	-0.127	0.952
1	28206319	G	A	C1orf38	chromosome 1 open reading frame 38	0.0001	+	+	17.71	NM_001105556:exon3:c.G400A:p.A134T	0.57	G§	0	N/A	N/A
1	28477192	T	C	PTAFR	platelet-activating factor receptor	0.0052	+	+	20.80	NM_001164721:exon3:c.A341G:p.N114S	0.57	G§	0	-0.506	0.867
1	33760820	G	A	ZNF362	zinc finger protein 362	0.0000	+		21.80	NM_152493:exon8:c.G1060A:p.A354	0.85	B§	1	0.336	0.784
1	36638206	G	A	MAP7D1	MAP7 domain containing 1	0.0011	+	+	34.00	NM_018067:exon4:c.G602A:p.R201Q	0.47	D§	0	0.157	0.792
1	111968011	G	A	OVGP1	oviductal glycoprotein 1, 120kDa	0.0000	+	+	12.85	NM_002557:exon4:c.C311T:p.T104I	0.57	G§	1	-0.023	0.988
1	177899689	C	A	SEC16B	SEC16 homolog B (S. cerevisiae)	0.0010	+	+	21.60	NM_033127:exon25:c.G3102T:p.Q1034H	0.87	C	0	N/A	N/A
1	197072434	T	A	ASPM	asp (abnormal spindle) homolog, microcephaly associated (Drosophila)	0.0013	+		14.55	NM_018136:exon18:c.A5947T:p.M1983L	0.57	G§	1	1.195	0.642
1	204418411	C	T	PIK3C2B	phosphoinositide-3-kinase, class 2, beta polypeptide	0.0007	+	+	35.00	NM_002646:exon15:c.G2248A:p.G750S	0.57	G§	1	-0.505	0.672
1	212799290	C	A	FAM71A	family with sequence similarity 71, member A	0.0000	+		13.78	NM_153606:exon1:c.C1071A:p.S357R	0.57	G§	1	N/A	N/A
1	228290051	T	G	C1orf35	chromosome 1 open reading frame 35	0.0093	+		21.10	NM_024319:exon5:c.A407C:p.E136A	-0.29	A	0	-0.079	0.934
2	10186509	C	T	KLF11	Kruppel-like factor 11	0.0003	+	+	14.69	NM_001177718:exon2:c.C224T:p.P75L	1.41	A	0	-0.129	0.892
2	55825844	A	G	SMEK2	SMEK homolog 2, suppressor of mek1 (Dictyostelium)	0.0026	+	+	23.90	NM_001122964:exon4:c.T629C:p.F210S	1.43	E	0	-0.222	0.631
2	73718061	A	G	ALMS1	Alstrom syndrome 1	0.0000	+	+	12.02	NM_015120:exon10:c.A8972G:p.D2991G	1.13	D§	0	-0.264	0.749
2	74757348	T	C	HTRA2	HtrA serine peptidase 2	0.0030	+	+	11.98	NM_013247:exon1:c.T215C:p.L72P	1.43	E	0	0.267	0.595
2	161029157	G	C	ITGB6	integrin, beta 6	0.0001	+	+	17.45	NM_000888:exon6:c.C844G:p.L282V	-0.84	G	1	N/A	N/A
3	126137556	G	A	CCDC37	coiled-coil domain containing 37	0.0052	+		12.36	NM_182628:exon7:c.G589A:p.A197T	-0.84	G	2	N/A	N/A
3	180334458	C	T	CCDC39	coiled-coil domain containing 39	0.0026	+		20.70	NM_181426:exon18:c.G2432A:p.R811H	0.22	A	1	0.167	0.882
3	186508024	A	C	RFC4	replication factor C (activator 1) 4, 37kDa	0.0000		+	12.98	NM_002916:exon10:c.T903G:p.H301Q	0.83	F	1	0.125	0.906
4	106158134	C	T	TET2	tet oncogene family member 2	0.0000	+	+	12.41	NM_017628:exon3:c.C3035T:p.P1012L	0.57	G§	1	-0.231	0.878
*4	106639176	T	A	GSTCD	glutathione S-transferase, C-terminal domain containing	0.0047	+		22.90	NM_001031720:exon2:c.T406A:p.C136S	0.57	G§	1	-0.199	0.781
5	11018087	T	C	CTNND2	catenin (cadherin-associated protein), delta 2 (neural plakophilin-related arm-repeat protein)	0.0000	+		25.80	NM_001332:exon18:c.A3083G:p.K1028R	-0.29	A	0	-1.940	0.401
5	140801897	C	T	PCDHGA11	protocadherin gamma subfamily A, 11	0.0007		+	18.54	NM_018914:exon1:c.C1103T:p.A368V	0.57	G	1	-0.587	0.624
5	140955835	C	T	DIAPH1	diaphanous homolog 1 (Drosophila)	0.0007	+		36.00	NM_005219:exon14:c.G1423A:p.E475K	0.57	G	1	0.344	0.612
5	149901055	G	A	NDST1	N-deacetylase/N-sulfotransferase (heparan glucosaminyl) 1	0.0036	+		18.54	NM_001543:exon2:c.G239A:p.R80H	1.43	E	0	-0.157	0.806
5	157053610	T	C	SOX30	SRY (sex determining region Y)-box 30	0.0013	+		15.84	NM_178424:exon5:c.A2000G:p.N667S	0.83	F	0	N/A	N/A
6	13316909	G	T	TBC1D7	TBC1 domain family, member 7	0.0042	+	+	23.60	NM_001143965:exon5:c.C413A:p.A138D	0.86	G§	1	-0.372	0.758
6	149856802	C	T	PPIL4	peptidylprolyl isomerase (cyclophilin)-like 4	0.0000	+	+	34.00	NM_139126:exon5:c.G394A:p.G132S	-0.29	A	0	0.100	0.900
6	159420630	A	T	RSPH3	radial spoke 3 homolog (Chlamydomonas)	0.0002	+	+	15.37	NM_031924:exon1:c.T379A:p.C127S	0.57	G	1	-0.140	0.858
6	167709705	G	A	UNC93A	unc-93 homolog A (C. elegans)	0.0052	+		24.10	NM_001143947:exon3:c.G455A:p.G152D	0.85	B	1	N/A	N/A
6	168317794	A	C	MLLT4	myeloid/lymphoid or mixed-lineage leukemia (trithorax homolog, Drosophila); translocated to, 4	0.0000	+	+	26.90	NM_001207008:exon18:c.A2522C:p.K841T	0.57	G§	1	-0.150	0.884
8	72958750	T	A	TRPA1	transient receptor potential cation channel, subfamily A, member 1	0.0000	+		14.64	NM_007332:exon17:c.A2059T:p.N687Y	-0.96	G	0	N/A	N/A
9	21166077	T	C	IFNA21	interferon, alpha 21	0.0002		+	10.42	NM_002175:exon1:c.A535G:p.K179E	1.12	D§	0	N/A	N/A
9	35404008	G	A	UNC13B	unc-13 homolog B (C. elegans)	0.0006	+	+	34.00	NM_006377:exon39:c.G4754A:p.R1585H	0.83	F	1	-0.377	0.658
10	13240791	C	A	MCM10	minichromosome maintenance complex component 10	0.0049	+		17.17	NM_018518:exon16:c.C2222A:p.T741K,	0.85	B	1	1.318	0.563
10	47087309	G	C	PPYR1	pancreatic polypeptide receptor 1	0.0000	+	+	15.45	NM_005972:exon3:c.G526C:p.A176P	0.57	G§	1	N/A	N/A
10	82187167	G	A	C10orf58	chromosome 10 open reading frame 58	0.0013	+		36.00	NM_032333:exon5:c.G491A:p.R164Q	0.56	G	1	N/A	N/A
10	105218301	C	G	CALHM1	calcium homeostasis modulator 1	0.0001	+		16.88	NM_001001412:exon1:c.G208C:p.V70L	-0.29	A	1	N/A	N/A
10	105727572	C	G	SLK	FYN oncogene related to SRC, FGR, YES	0.0000	+	+	20.60	NM_014720:exon1:c.C69G:p.H23Q	-0.29	A	1	0.182	0.774
ǂ10	105797397	G	A	COL17A1	collagen, type XVII, alpha 1	0.0005		+	14.75	NM_000494:exon46:c.C3205T:p.R1069W	0.57	G	1	N/A	N/A
10	105893436	T	G	WDR96	WD repeat domain 96	0.0005	+		23.90	NM_025145:exon35:c.A4538C:p.D1513A	-0.29	A	1	0.048	0.988
11	400124	C	G	PKP3	plakophilin 3	0.0013	+	+	12.37	NM_007183:exon6:c.C1431G:p.N477K	-0.71	G§	0	N/A	N/A
11	73074872	G	A	ARHGEF17	Rho guanine nucleotide exchange factor (GEF) 17	0.0003	+	+	18.47	NM_014786:exon16:c.G5327A:p.C1776Y	1.13	D§	0	0.162	0.931
11	108277861	C	T	C11orf65	chromosome 11 open reading frame 65	0.0064	+	+	21.30	NM_152587:exon4:c.G190A:p.A64T	1.13	C	1	N/A	N/A
11	124742851	G	A	ROBO3	roundabout, axon guidance receptor, homolog 3 (Drosophila)	0.0004	+	+	20.20	NM_022370:exon9:c.G1402A:p.V468M	1.31	A	0	-0.019	0.993
11	126147035	T	G	FOXRED1	FAD-dependent oxidoreductase domain containing 1	0.0013	+	+	18.40	NM_017547:exon10:c.T1171G:p.L391V	-0.58	F	1	-0.152	0.815
ǂ12	2968094	G	T	FOXM1	forkhead box M1	0.0000	+	+	13.37	NM_202003:exon8:c.C1957A:p.P653T	0.29	D§	0	0.885	0.615
*12	12630140	T	G	DUSP16	dual specificity phosphatase 16	0.0026	+		16.34	NM_030640:exon7:c.A1625C:p.D542A	-0.69	B§	2	-0.324	0.686
*12	49498284	T	G	LMBR1L	limb region 1 homolog (mouse)-like	0.0040	+		16.10	NM_018113:exon5:c.A382C:p.M128L	0.83	F	2	0.156	0.824
12	56335802	T	C	DGKA	diacylglycerol kinase, alpha 80kDa	0.0000		+	17.40	NM_001345:exon16:c.T1271C:p.V424A	1.11	D	0	0.551	0.544
*12	96374381	C	A	HAL	histidine ammonia-lyase	0.0006	+	+	25.70	NM_002108:exon17:c.G1472T:p.G491V	1.14	D§	1	-0.479	0.922
12	126139069	C	T	TMEM132B	transmembrane protein 132B	0.0002	+	+	10.88	NM_052907:exon9:c.C3050T:p.S1017L	1.14	D	0	-2.626	0.023
15	75014793	T	A	CYP1A1	cytochrome P450, family 1, subfamily A, polypeptide 1	0.0003	+	+	14.09	NM_000499:exon2:c.A646T:p.S216C	0.83	F	1	N/A	N/A
16	449449	G	A	NME4	non-metastatic cells 4, protein expressed in	0.0000		+	11.74	NM_005009:exon3:c.G296A:p.R99H	0.85	B§	1	-0.076	0.943
*16	2133701	G	A	TSC2	tuberous sclerosis 2	0.0040	+		12.84	NM_001114382:exon32:c.G3820A:p.A1274T	0.85	B§	1	-0.229	0.658
16	11785220	G	A	TXNDC11	thioredoxin domain containing 11	0.0014	+		18.28	NM_015914:exon8:c.C1826T:p.A609V	0.85	B§	1	0.134	0.896
16	20796338	G	A	ACSM3	acyl-CoA synthetase medium-chain family member 3	0.0013	+	+	22.00	NM_005622:exon8:c.G1052A:p.S351N	0.57	G	0	0.751	0.496
16	53321892	A	G	CHD9	chromodomain helicase DNA binding protein 9	0.0076		+	18.22	NM_025134:exon27:c.A5213G:p.K1738R	0.65	G	0	0.095	0.910
17	5425076	A	G	NLRP1	NLR family, pyrin domain containing 1	0.0042		+	10.35	NM_033007:exon12:c.T3461C:p.M1154T	-0.56	D§	0	0.293	0.727
17	48762223	G	A	ABCC3	ATP-binding cassette, sub-family C (CFTR/MRP), member 3	0.0013	+	+	22.70	NM_003786:exon29:c.G4267A:p.G1423R	0.85	B§	0	-0.043	0.994
17	61432613	T	A	TANC2	tetratricopeptide repeat, ankyrin repeat and coiled-coil containing 2	0.0000	+	+	25.00	NM_025185:exon12:c.T2222A:p.F741Y	0.85	B§	0	-0.213	0.859
19	11598418	G	A	ZNF653	zinc finger protein 653	0.0000		+	16.16	NM_138783:exon4:c.C860T:p.A287V	1.41	A	1	0.168	0.829
19	13226094	G	A	TRMT1	TRM1 tRNA methyltransferase 1 homolog (S. cerevisiae)	0.0002	+	+	20.70	NM_017722:exon4:c.C640T:p.R214W	1.41	A	1	0.222	0.737
19	57175814	C	G	ZNF835	zinc finger protein 835	0.0009	+		18.91	NM_001005850:exon2:c.G753C:p.E251D	0.86	G	1	-0.797	0.556
19	57723459	C	T	ZNF264	zinc finger protein 264	0.0000	+	+	11.70	NM_003417:exon4:c.C994T:p.R332W	0.86	G	1	-0.132	0.882
20	44463002	A	G	SNX21	sorting nexin family member 21	0.0000	+		22.20	NM_152897:exon2:c.A184G:p.S62G	0.85	B§	1	-0.220	0.797
6	153312343	TTTTA	T	MTRF1L	mitochondrial translational release factor 1-like	0.0000	NA	+ (SIFT-INDEL)	14.77	NM_019041:exon6:c.915_918del:p.305_306del	0.57	G	1	-0.095	0.924

Ref = reference allele, Alt = alternate allele. Alt Freq = alternate allele frequency (consensus frequency for the alternate allele from 1000 Genomes and/or Exome Sequencing Project, as described in the Methods), LOD = maximum LOD score for linkage markers found within a 10Mb window of the sequencing variant, Unaff = number of sequenced unaffected individuals who carry the variant, logFC = log fold change of expression differential (N/A indicates no expression data is available for the gene), FDR = false discovery rate-adjusted p-value. All variants are predicted to be non-synonymous exonic variants except the deletion at the end of the Table. A plus sign (+) denotes a damaging prediction. For variants segregating in families B, D, or G, a (§) indicates that variant was also shared by an individual in the same family with a probable or possible IA or an abdominal aortic aneurysm.

Of the 68 retained variants, five variants (found in the genes *GSTCD*, *DUSP16*, *LMBR1L*, *HAL*, and *TSC2*) were found in definite IAs in two families; in all of these cases, the variant segregated fully with definite IA in only one family. Two other variants (found in the genes *COL17A1* and *FOXM1*) were the only variants of the 68 retained variants that were labeled with vascular-related Gene Ontology annotations (i.e. GO:0005604 basement membrane and GO:0005581 collagen; and GO:0001570 vasculature development and GO:0001570 vasculogenesis; respectively).

### Linkage

The distribution of genome-wide LOD scores for each family is depicted in Figs. S1, S2, S3, S4, S5, S6, and S7, with the WES variants satisfying biological filters 1–6 superimposed. The maximum possible LOD score for each family given the model parameters and the specific pedigree structure is also reportedThe highest LOD score obtained by linkage markers within a 10Mb window centered on each sequencing variant is recorded in Table 4. Of the 68 WES variants satisfying biological filters 1–6 and manual inspection, 23 variants had a LOD score for a linkage marker within 10Mb of the sequencing variant that fell within 0.01 of the highest possible LOD score for that family.

The 23 variants within a possible linkage peak were distributed among all families except family F, where the highest LOD score for a linkage marker within 10Mb of a filtered sequencing variant was 0.83 but the highest possible LOD score for the family was 1.12. Family B had the most retained variants within possible linkage peaks (n = 9); followed by family D (n = 4); families A, E, and G (n = 3); and family C (n = 1). Of the 23 variants, only 8 also met the optional prioritization criteria of segregating with all aneurysmal phenotypes and not being carried by an unaffected individual (*KLF11* variant in family A, variants in *ABCC3* and *TANC2* in family B, variants in *ALMS1* and *ARHGEF17* in family D, and variants in *SMEK2*, *HTRA2*, *and NDST1* in family E).

While none of the 68 variants coincided with well-established GWAS association signals, 6 of the variants were found within IA linkage peaks identified in previously published family studies, independent of the families in this report. Four variants (found in the genes *C1orf38*, *PTAFR*, *ZNF362*, and *MAP7D1*) fell within the linkage peak 1p34.3–36.13 [7, 8], while 2 variants (found in the genes *ROBO3* and *FOXRED1*) were located in the linkage peak 11q24–25 [10–12]. None of these 6 genes were suggested as candidate genes by the authors of the published linkage studies. The linkage regions each cover hundreds of genes, as they span approximately 24 and 14 Mb respectively.

### RNA Expression

Expression data was obtained in 51 of the 68 candidate genes in an independent set of IA cases and controls. Log fold changes and FDR-adjusted p-values for each gene is displayed in Table 4. Only 1 gene (*TMEM132B*) of the 51 genes showed differential expression (overexpressed with log fold change = 2.63, FDR-adjusted p-value = 0.023).

## Discussion

### TMEM132B

Exome sequencing presents an opportunity to explore the contribution of rare variation to complex disorders like IA. We have used this approach to identify 68 rare variants in 68 genes that segregate within 7 densely affected families. Of the 51 genes that were expressed in IA tissue, one gene (*TMEM132B*) was found to be significantly overexpressed in IA tissue in comparison to control vascular tissue.

*TMEM132B*, or transmembrane protein 132B, is a relatively uncharacterized protein of unknown function. The variant segregating in the family is rare (0.024% frequency in European American samples in the Exome Sequencing Project and not found in 1000 Genomes) and predicted damaging by SIFT, PolyPhen-2, and CADD due to a change from the polar amino acid serine to the nonpolar amino acid leucine at a highly conserved position. Each of the individuals with a definite IA in family D was heterozygous for the variant. Mutations inherited in a dominant manner often lead to a disease phenotype through a gain of function mechanism, which would be supported by the overexpression of *TMEM132B* in IA tissue as compared to control vessels. It is also possible, however, that dominantly-inherited mutations exert their effect via haploinsufficiency or dominant negative mechanisms. Further studies are required to confirm the role of *TMEM132B* in IA and through what mechanism the variant identified in this study may act.

The *TMEM132B* variant was not inherited by individual 11 in family D. Individual 11 was diagnosed as a possible IA due to the presence of a small aneurysm identified through non-invasive imaging (i.e. 1–2mm, verified by 3 independent neurologists). This is in contrast to the definite IAs clearly identified in this individual’s sibling and cousins; thus, individual 11 is most likely actually unaffected.

### Prioritization of Variants within Families

Expression information was only available for 51 of the 68 candidate genes; thus, RNA expression cannot confirm or rule out the role of the remaining 17 genes in IA pathophysiology. Additionally, a subset of the other 50 variants with expression data may also contribute to IA in ways not captured by the RNA expression experiment and should be explored. In order to further study the cause of IA in each of the remaining families, candidate variants in each family must be prioritized.

In families C and E, segregation analysis reduced the number of prioritized variants to only 2 and 4 variants, respectively. For family C, the two variants have a CADD score >20. The variant in *SEC16B* is not found within a potential linkage peak; however, in support of its potential significance in disease susceptibility, it is not carried by any tested unaffected family member. The variant in *C11orf65*, on the other hand, is found within a potential linkage peak but is also inherited by an unaffected family member. For family E, three variants segregate in the family (and a fourth variant in *GSTCD* is found in only one individual in family E but segregates fully in family G). The three variants that segregate in the family (in genes *SMEK2*, *HTRA2*, and *NDST1*) all are found within potential linkage peaks. Data are not available from any unaffected family members. Therefore, further prioritization among these three variants could incorporate CADD scores, which range from 11.98 for the *HTRA2* variant to 23.9 for the *SMEK2* variant.

### Considerations for Pedigree and Phenotypic Data

It is possible that genetic heterogeneity, phenocopies, or gene-environment interactions could explain one or more IAs in the families chosen for this study. In this case, the criterion requiring all affected individuals to share a variant would miss important disease-contributing variants. Similar family-based sequencing studies in the future could relax this segregation criterion with the recognition that a much larger number of variants will be retained. Family-based aggregative association tests that incorporate different penetrance models could also be employed with a larger number of samples.

The availability and quality of clinical data is also critical to consider in complex disease WES studies. In this study, several families also had individuals with probable and possible IAs (see Table 1 for phenotype definitions), and one family also had an occurrence of an abdominal aortic aneurysm (Fig. 1). Given the high density of definite IAs in these families, it is likely that some or all of the probable and possible IAs have disease due to the same disease-contributing variant. Additionally, given the possible genetic link between different forms of aneurysms [37], the abdominal aortic aneurysm may also share the same genetic etiology within that family. We thus flagged variants that segregated fully among all individuals with an aneurysm (definite, probable, or possible IA, or an abdominal aortic aneurysm) (Tables 2–3). This represents a possible method for prioritizing variants for further study, with the caveat that including non-definite IAs increases the likelihood of genetic heterogeneity, phenocopies, and gene-environment interactions.

Another approach to prioritize variants for further study is to utilize genotypic data from unaffected individuals. The ability of this approach to rapidly narrow down the number of variants under consideration is readily apparent from this study (Tables 2–3), but there are major concerns about inflating false negative rates by using unaffected individuals. Given the traditionally late age of onset for intracranial aneurysms, only individuals who had an MRA confirming the absence of IA at age 45 or older were sequenced as unaffected samples. Despite these precautions, the unaffected individuals in this study were still relatively close in age to the age at diagnosis of their relatives who had an IA, and it is possible that the unaffected individuals will actually develop an IA later in life due to a genetic predisposition.

The difficulty in defining an unaffected also surfaces when considering the putative obligate carriers in these families. In Family A, individual A-7 also had an MRA done at age 64 that excluded the presence of an IA, yet we would posit that this individual likely passed a causative genetic variant to her daughter (A-10), whose IA is more likely to have a genetic basis due to her young age of onset. Without the daughter’s data, individual A-7 would have likely been chosen as an unaffected individual for sequencing, especially given that she had major environmental risk factors (a history of smoking and hypertension). In Family E, the sequenced individual E-9 is also an obligate carrier under our model. Unlike individual A-7, an MRA could not be obtained on individual E-9, and she did not have a history of smoking or hypertension. Since all affected individuals in family E had at least one environmental risk factor and individual E-9 did not, it is possible that the causative genetic variant in family E requires an additional environmental insult to lead to IA development. The importance of strong environmental risk factors such as smoking to the development of aneurysms, even in the context of rare causal genetic variants, cannot be underestimated. Alternative methods of prioritization of variants that incorporate this possibility should be explored. Thus, unaffected status in this study was used as a mechanism for possible prioritization but not for automatic exclusion of variants. The ability to use unaffected individuals will vary in studies of different diseases and will likely be more fruitful in those diseases that appear to have a smaller environmental/lifestyle contribution.

For future family-based sequencing studies in complex disease, it may not be feasible to sequence as many individuals per pedigree as was done for this study. Thus, it is critical to carefully select samples based on the quality of phenotyping and the pedigree structure. Recently developed tools offer statistical methods to select related subjects for sequencing based on genetic distance [38], samples that span multiple generations (Exome Picks, http://genome.sph.umich.edu/wiki/ExomePicks), and a combination of both of these methods [39]. As evident from Tables 2–3, selecting families with more closely related individuals, such as families with full siblings as in Families F and G, will yield a smaller number of initially called variants across the family. Yet, the power to narrow down the number of variants segregating with disease is diminished in such families due to the naturally larger percentage of alleles shared, as compared to families with individuals in multiple generations such as in Family C. Thus, where possible, selection of more distantly related family members for sequencing studies will have greater power to generate a narrowed list of prioritized variants.

For some families, it may be possible to combine linkage and sequencing data to find causative variants. The families sequenced in this study were included as a part of a larger linkage study reported previously [9]. The same model parameters used for WES variant filtering was applied for multipoint linkage analysis. Since any given marker may have been uninformative for a family, a maximum LOD score was reported within a 10Mb window of the sequence variant’s chromosomal position. Although only modest evidence of linkage was obtained, several sequencing variants lay within the linkage regions in these families (S1, S2, S3, S4, S5, S6, and S7 Figs.). Many variants, however, did not overlap with any evidence of linkage, suggesting that these families were either not fully informative for robust linkage analysis near these loci, or the sequencing variants identified are not causative genetic variants in these families.

### Considerations for Exonic Variation

In recent years, WES has emerged as a practical method for systemically exploring rare coding variation. Since the majority of known genetic causes of Mendelian disorders affect protein coding regions [40], the exome is a logical starting place to identify potentially causative variants in diseases that exhibit Mendelian inheritance. The densely-affected families sequenced in this study appear to display autosomal dominant inheritance; therefore, we hypothesized that coding variants may explain most or even all of these cases.

Due to imperfect capture and alignment, WES generates some off-target, non-exonic variant calls. While it is possible that important variation exists in these off-target regions, a higher percentage of calls in these regions are of poorer quality. Thus, only those variants within exonic or splicing regions were retained in this experiment. Since different databases contain different numbers of and boundaries for genes and exons [41], a consensus prediction of gene and exon boundaries was made to determine those variants that fell within exonic or splicing regions. In order to minimize the type I error rate by using functional predictions of the highest confidence, the intersection of functional predictions from three different databases (RefSeq, UCSC, and Ensembl) was used for this study. Thus, variants were only retained if they were predicted by all three databases to be within exonic or splicing regions. Other WES studies may choose to generate a larger set of variants by prioritizing all variants in the union rather than the intersection of functional predictions from multiple databases; however, appropriate methods for validating variants with functional predictions that differ by database should be employed.

It is possible that non-coding variants and/or epistatic interactions are important in IA development in these families and in other complex diseases, in which case alternate study designs should be utilized. At the time of this study, whole genome sequencing could have only been employed at the expense of sequencing fewer individuals, and annotations and bioinformatics tools available for non-coding sequence were less robust. Given that whole genome sequencing generates about 3 million SNVs per genome [42], annotations and bioinformatics tools are even more critical for practical prioritization of candidate variants. In the future, techniques like whole genome sequencing, as well as targeted resequencing, transcriptome sequencing, and other high throughput study designs, can be applied to fully catalogue the role of genetic variation in IA development.

### Considerations for Allele Frequency

The average individual has around 15,000 exonic single nucleotide variants (SNVs) differing from the reference human genome sequence [24]. In order to narrow down the number of variants identified by a WES study, initial studies [43, 44] focused on rare diseases and limited analysis to novel variants. This strategy is too restrictive for more common diseases such as IA. In the particular subset of families used for this study, there is a uniquely high incidence of IA, which enriches for the possibility of identifying rare, highly penetrant variants of larger effect sizes. Rare variants and less common variants are typically defined as less than 1% and 1–5% minor allele frequency, respectively [45, 46]. Given the rarity of families that are as densely affected as the ones in this study, a 1% minor allele frequency threshold was set. It is possible, however, that a variant of higher minor allele frequency causes IA in one or more of these families. Future studies with a much larger sample size could employ aggregative association tests [47] with relaxation of the allele frequency threshold.

In this study, allele frequencies specifically from European American populations were available from public databases. Given that rare variants can be population-specific [48], the selection of appropriate allele frequency databases is critical. In lieu of publicly available allele frequencies, future studies may consider sequencing a large number of internal controls and possibly requesting commonly available controls to sequence as well. While not feasible for the current study, such a design would help control for platform- and pipeline-specific artifacts in sequencing while ensuring phenotyping quality for controls.

While it is standard for WES studies to utilize public databases to filter variants, it is also valuable to use internal frequency databases that are specific to the sequencing and variant calling pipeline. Because variant calling can be lab-specific due to the technology used, in this study variants were annotated for binned minor allele frequencies from 290 unrelated samples without a known cardiovascular phenotype that were exome sequenced at CIDR. Thus variants that would have otherwise been considered rare or novel when compared against public databases, but that were actually a recurring artifact of the sequencing, were captured as having a high CIDR binned minor allele frequency. Given that the bioinformatics pipeline used in this study differed slightly from that of the internal database, the internal database filter may have missed some artifacts specific to the variant calling method. Variants that were monomorphic (i.e. all heterozygous or homozygous for the alternate allele) across all samples were also removed since it is highly unlikely that the identical rare disease-causing allele would be shared by both affected and unaffected individuals in multiple families.

Insertion/deletion allele frequencies in both internal and external databases are inherently less accurate than frequencies for SNVs, due to the increased difficulty and variation in calling structural variants. Also, differences in how position coordinates are assigned as well as reference and alternate allele designations further makes comparison challenging. The 26 insertion/deletions that passed biological filters 1–6 (described in the Methods) in all cases except for one were shared in almost all or all of the 7 families sequenced in this study. Just as variants that were monomorphic across all datasets were removed as probable sequencing or pipeline artifacts, it is very unlikely that any given rare disease-causing insertion/deletions would also be shared across all or almost all families in a complex disease. It is possible that multiple families may carry different disease-causing insertion/deletions in the same gene, but this pattern was not seen. Thus, a second internal frequency comparison set of 500 samples that had a more similar bioinformatics pipeline to the IA samples sequenced in this study (i.e. use of GATK Unified Genotyper for variant calling) was used for manual review in combination with IGV visual inspection for the 26 insertion/deletions remaining after application of biological filters. Manual review as described in the Methods excluded all but one of the 26 insertion/deletions, demonstrating that manual inspection and use of an internal dataset generated by a similar bioinformatics pipeline are critical for reviewing insertion/deletions in sequencing experiments. Future studies may also consider utilizing newer local re-assembly-based methods for variant calling, such as FreeBayes [49] or GATK’s HaplotypeCaller, which may improve the accuracy of insertion/deletion calls.

### Consideration for Functional Predictions of Exonic Variation

More severe amino acid substitutions are more likely to present clinically [40], so most WES studies to date have focused on non-synonymous SNVs and insertion/deletions. In this study, we also opted to focus on these variants, as predicted by the intersection of the three gene databases (RefSeq, UCSC, and Ensembl). Future studies focused on exonic variation could also study the effect of synonymous variation, which has been shown to also play an important role in human disease [50]. At the time of this study, fewer validated tools existed to examine the role of synonymous variation in sequencing data.

In this study, several programs were used to measure the level of conservation of a locus and the predicted pathogenicity of a variant. The programs have varying degrees of sensitivity and specificity for different kinds of variants, particularly due to the use of different but not completely independent data sources when generating predictions [51]. The bioinformatics community is working to develop tools that will be able to better integrate information to provide a more informed pathogenicity prediction. One such tool, the CADD program [31], was recently introduced but has not been applied to a large number of datasets. Since there are few published studies implementing CADD, we have conservatively removed only variants with a C-score <10, thus retaining variants that are predicted by CADD to be among the 10% most deleterious substitutions in the human genome.

### Considerations for Biological Processes and Pathways

The filtering schema did not employ any assumptions about biological processes or pathways. Variants were annotated for GO terms chosen for possible relation to IA formation; however, only two variants in the final candidate variant list (variants found in the genes *COL17A1* and *FOXM1*) had one or more of these GO annotations. While using GO annotations as a filter is a powerful method for narrowing a list of variants, such an approach would depend on the comprehensiveness of GO annotations, as well as the reliability of investigator-chosen GO terms. To avoid subjectivity in selecting biological processes or pathways, future studies with larger sample sizes should consider employing formal gene set enrichment analysis, which eliminates the need to choose pathways *a priori*. Even for smaller datasets, use of GO annotations may help determine which gene variants to pursue first in additional experiments to explore possibly causal associations between the variant and disease of interest.

### Summary and Future Directions

This is one of the few studies published to date that apply WES in a cohort of well-characterized families densely affected with a common complex disease without an *a priori* focus on a particular pathway or genomic region. We have laid out many considerations for future WES studies in complex disease, including the use of pedigree and phenotypic data, defining gene and exon boundaries, sources for allele frequency estimates, proper interpretation of *in silico* functional predictions, the role of environmental factors in the determination of potentially causal rare variants, and the possible utility of combining pathway information with sequencing data.

In this study, 68 rare exonic variants in 68 genes were identified. Of these genes, one gene (*TMEM132B*) was significantly differentially expressed in IA versus control tissue. Further studies are needed to confirm and explore the *TMEM132B* variant, as well as the possible contribution of the other 67 variants. Replication and/or meta-analysis with similar sequencing studies using larger sample sizes could be used to gather further evidence for specific genes on this list. Additionally, a subset of these variants, which can be prioritized through any of the methods discussed in this study, could be explored through functional studies in models where vascular phenotypes can be easily observed, such as zebrafish. Targeted gene editing, such as through the CRISPR-Cas system, could help test whether a given variant disrupts the normal functioning of the relevant gene and whether such a disruption leads to a phenotype of interest. Ultimately, such a model should also enable investigation of whether the disrupted phenotype can be rescued by reintroduction of the wild type allele or interference with the variant allele. For comprehensive exploration of the variants identified in this study, multiple methods of experimental validation may be necessary. This study represents a necessary first step in the evaluation of role of rare variants in a common complex disease. Further evaluation in other familial and sporadic samples, as well as multi-ethnic samples, will be essential.

## Supporting Information

S1 Fig Summary of genome-wide multipoint linkage analysis for Family A. Details of the disease-specific modeling are described in the Methods. Positions of candidate single nucleotide variants and insertion/deletions identified in the whole exome sequencing data are denoted by diamonds and crosses, respectively.
(TIF)

S2 Fig Summary of genome-wide multipoint linkage analysis for Family B. Details of the disease-specific modeling are described in the Methods. Positions of candidate single nucleotide variants and insertion/deletions identified in the whole exome sequencing data are denoted by diamonds and crosses, respectively.
(TIF)

S3 Fig Summary of genome-wide multipoint linkage analysis for Family C. Details of the disease-specific modeling are described in the Methods. Positions of candidate single nucleotide variants and insertion/deletions identified in the whole exome sequencing data are denoted by diamonds and crosses, respectively.
(TIF)

S4 Fig Summary of genome-wide multipoint linkage analysis for Family D. Details of the disease-specific modeling are described in the Methods. Positions of candidate single nucleotide variants and insertion/deletions identified in the whole exome sequencing data are denoted by diamonds and crosses, respectively.
(TIF)

S5 Fig Summary of genome-wide multipoint linkage analysis for Family E. Details of the disease-specific modeling are described in the Methods. Positions of candidate single nucleotide variants and insertion/deletions identified in the whole exome sequencing data are denoted by diamonds and crosses, respectively.
(TIF)

S6 Fig Summary of genome-wide multipoint linkage analysis for Family F. Details of the disease-specific modeling are described in the Methods. Positions of candidate single nucleotide variants and insertion/deletions identified in the whole exome sequencing data are denoted by diamonds and crosses, respectively.
(TIF)

S7 Fig Summary of genome-wide multipoint linkage analysis for Family G. Details of the disease-specific modeling are described in the Methods. Positions of candidate single nucleotide variants and insertion/deletions identified in the whole exome sequencing data are denoted by diamonds and crosses, respectively.
(TIF)
