# Supplementary figures and images for: Lessons Learned from Whole Exome Sequencing in Multiplex Families Affected by a Complex Genetic Disorder, Intracranial Aneurysm
Source: PLoS One. 2015 Mar 24;10(3):e0121104. doi: 10.1371/journal.pone.0121104 (PMC4372548; doi:10.1371/journal.pone.0121104)

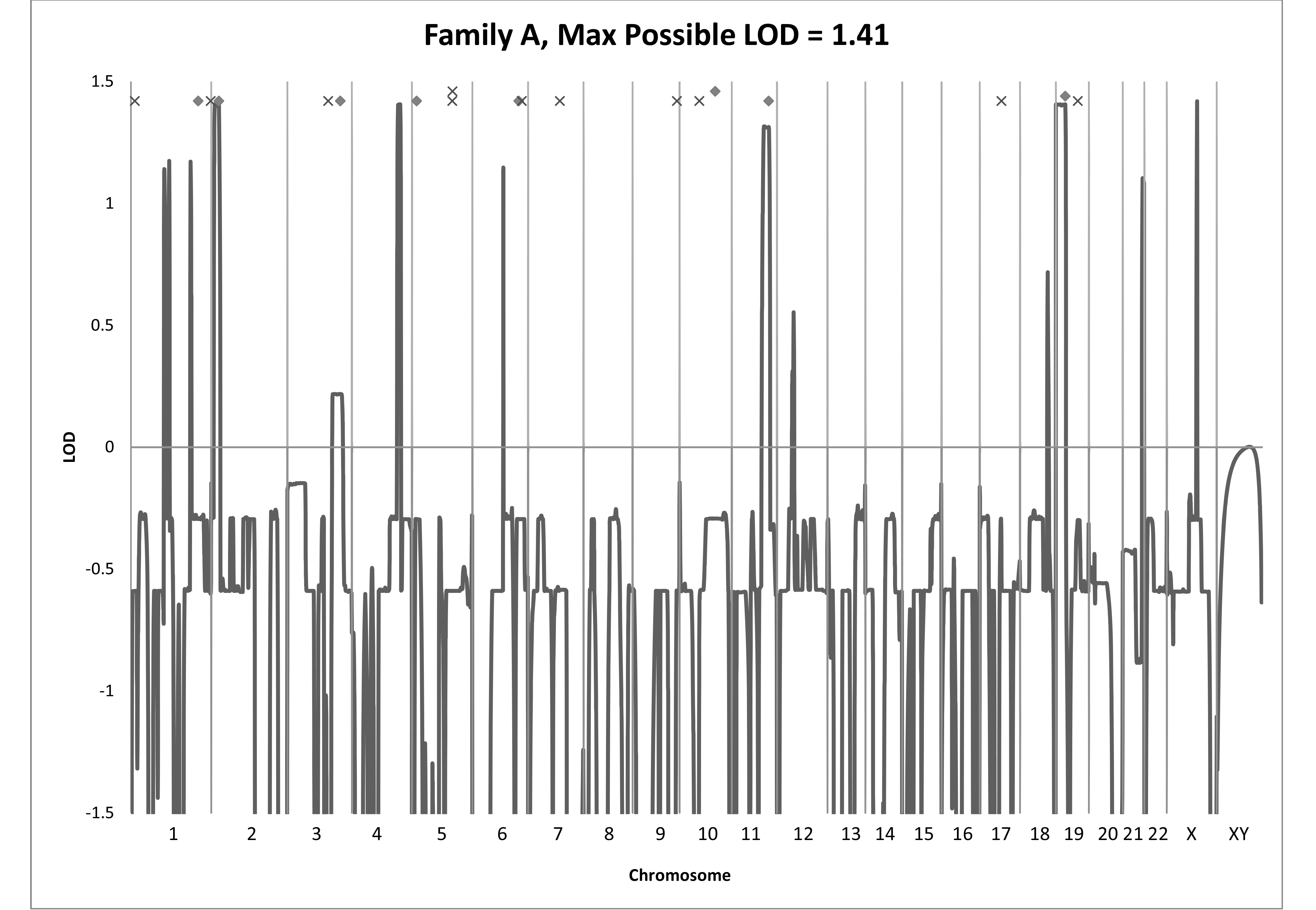

Supplement: S1 Fig — Details of the disease-specific modeling are described in the Methods. Positions of candidate single nucleotide variants and insertion/deletions identified in the whole exome sequencing data are denoted by diamonds and crosses, respectively. (TIF) [file pone.0121104.s001.tif]

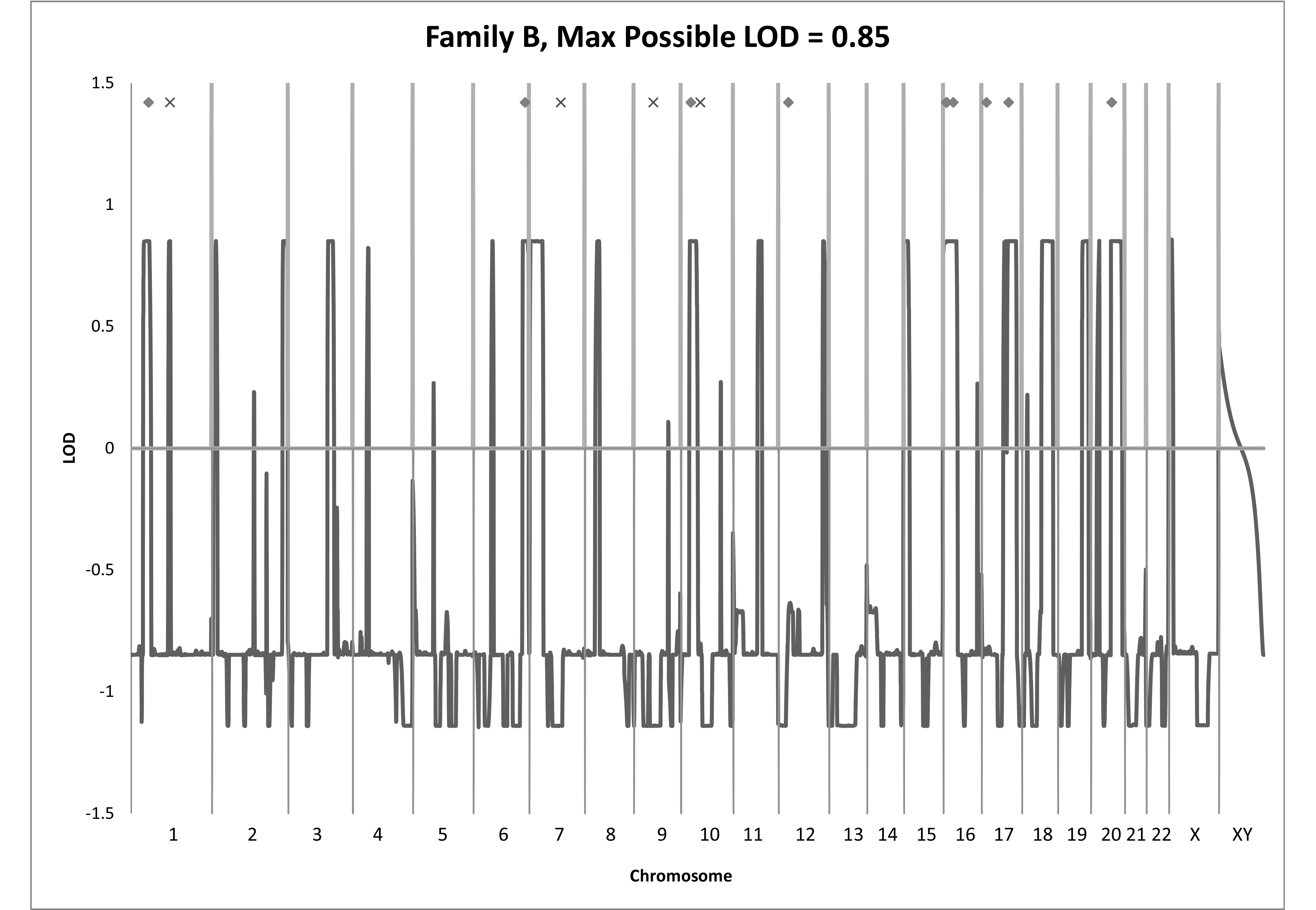

Supplement: S2 Fig — Details of the disease-specific modeling are described in the Methods. Positions of candidate single nucleotide variants and insertion/deletions identified in the whole exome sequencing data are denoted by diamonds and crosses, respectively. (TIF) [file pone.0121104.s002.tif]

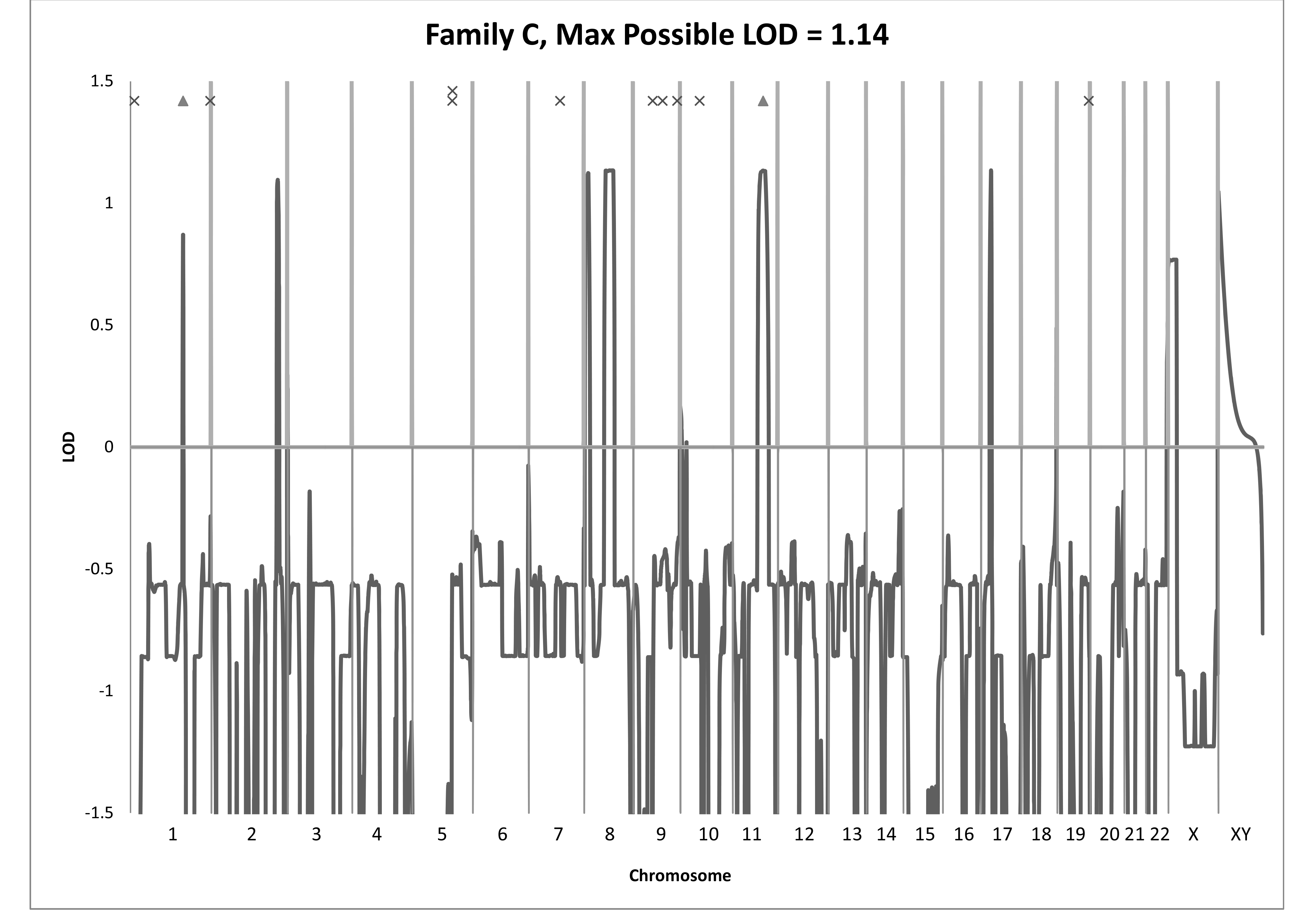

Supplement: S3 Fig — Details of the disease-specific modeling are described in the Methods. Positions of candidate single nucleotide variants and insertion/deletions identified in the whole exome sequencing data are denoted by diamonds and crosses, respectively. (TIF) [file pone.0121104.s003.tif]

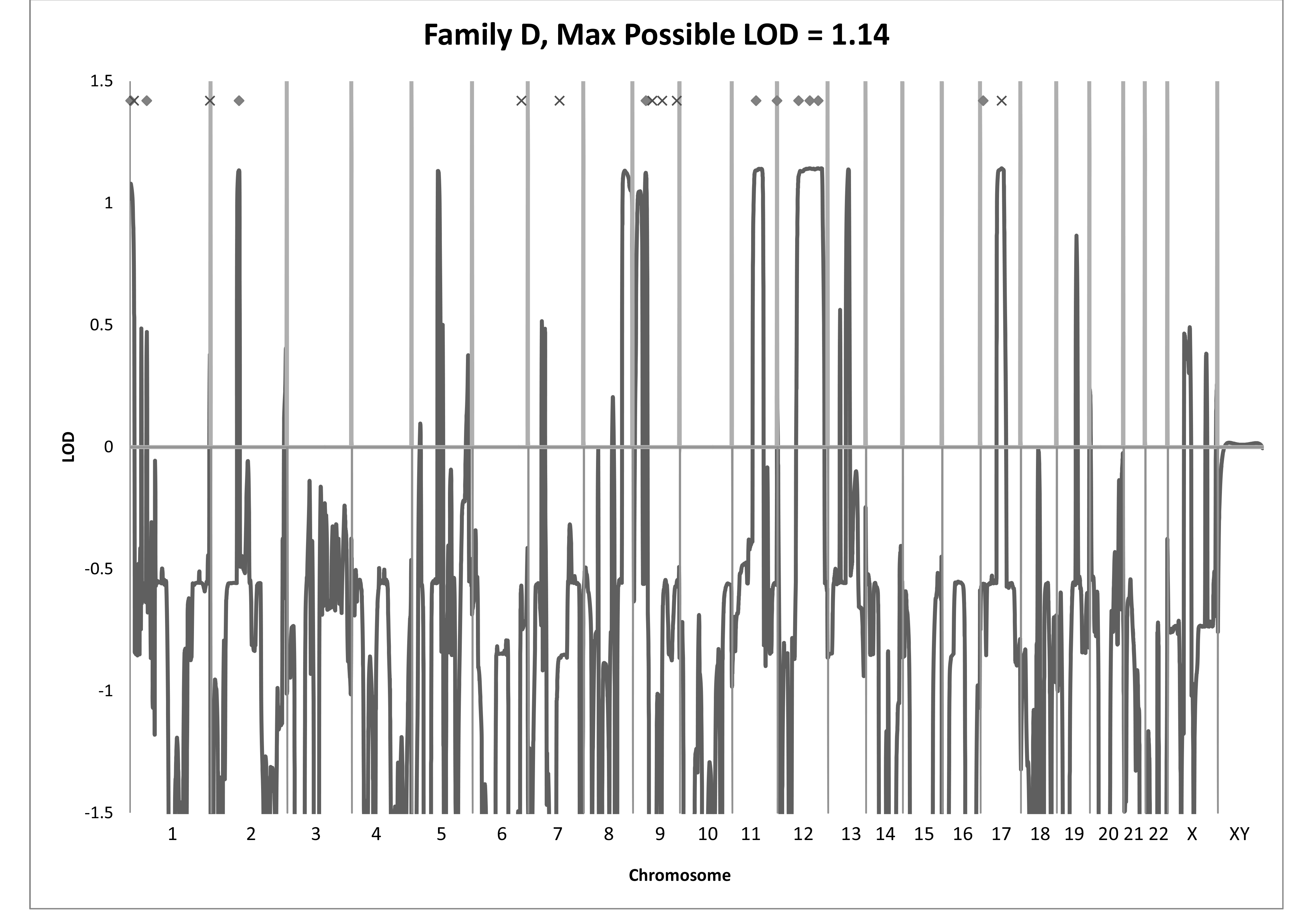

Supplement: S4 Fig — Details of the disease-specific modeling are described in the Methods. Positions of candidate single nucleotide variants and insertion/deletions identified in the whole exome sequencing data are denoted by diamonds and crosses, respectively. (TIF) [file pone.0121104.s004.tif]

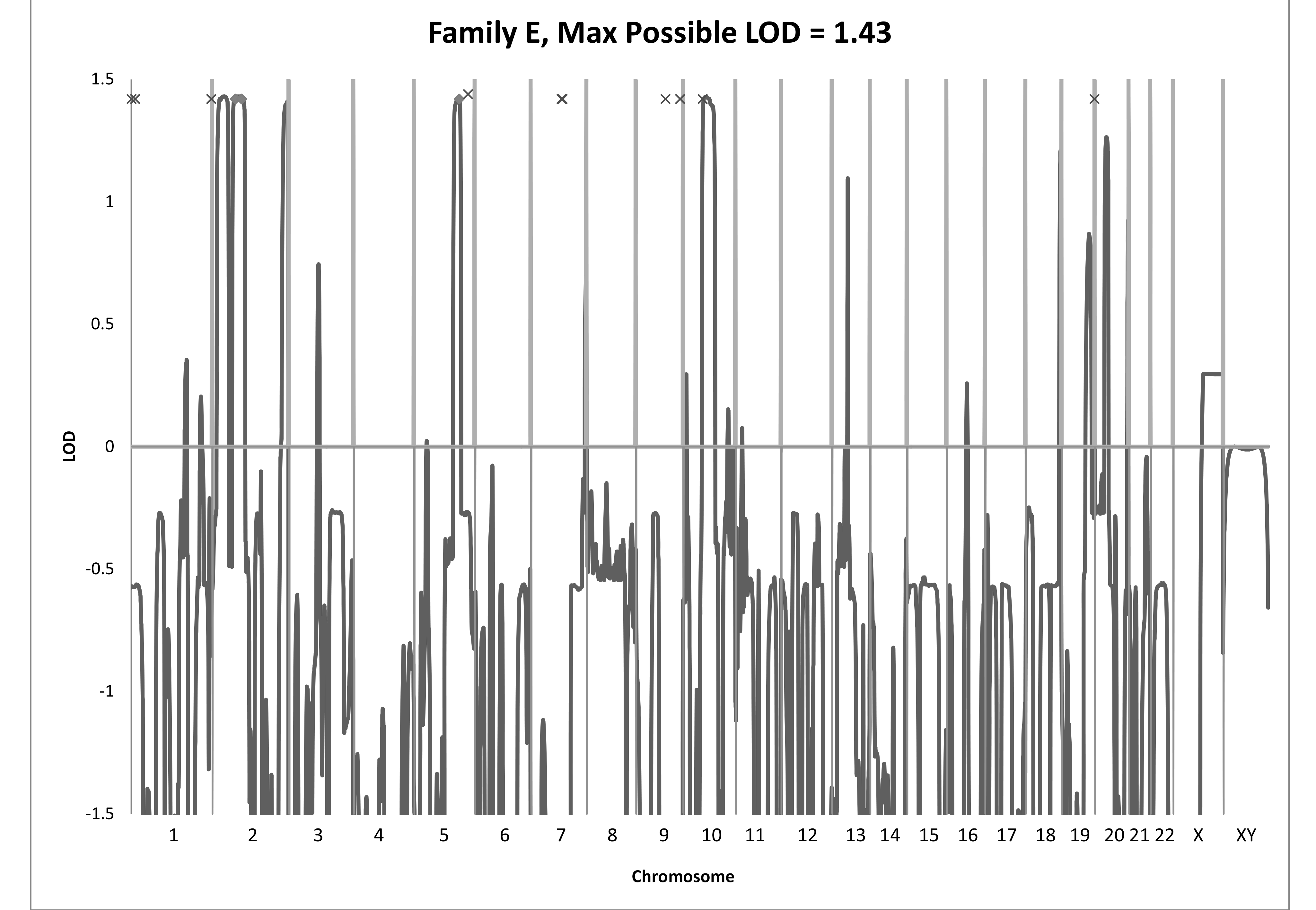

Supplement: S5 Fig — Details of the disease-specific modeling are described in the Methods. Positions of candidate single nucleotide variants and insertion/deletions identified in the whole exome sequencing data are denoted by diamonds and crosses, respectively. (TIF) [file pone.0121104.s005.tif]

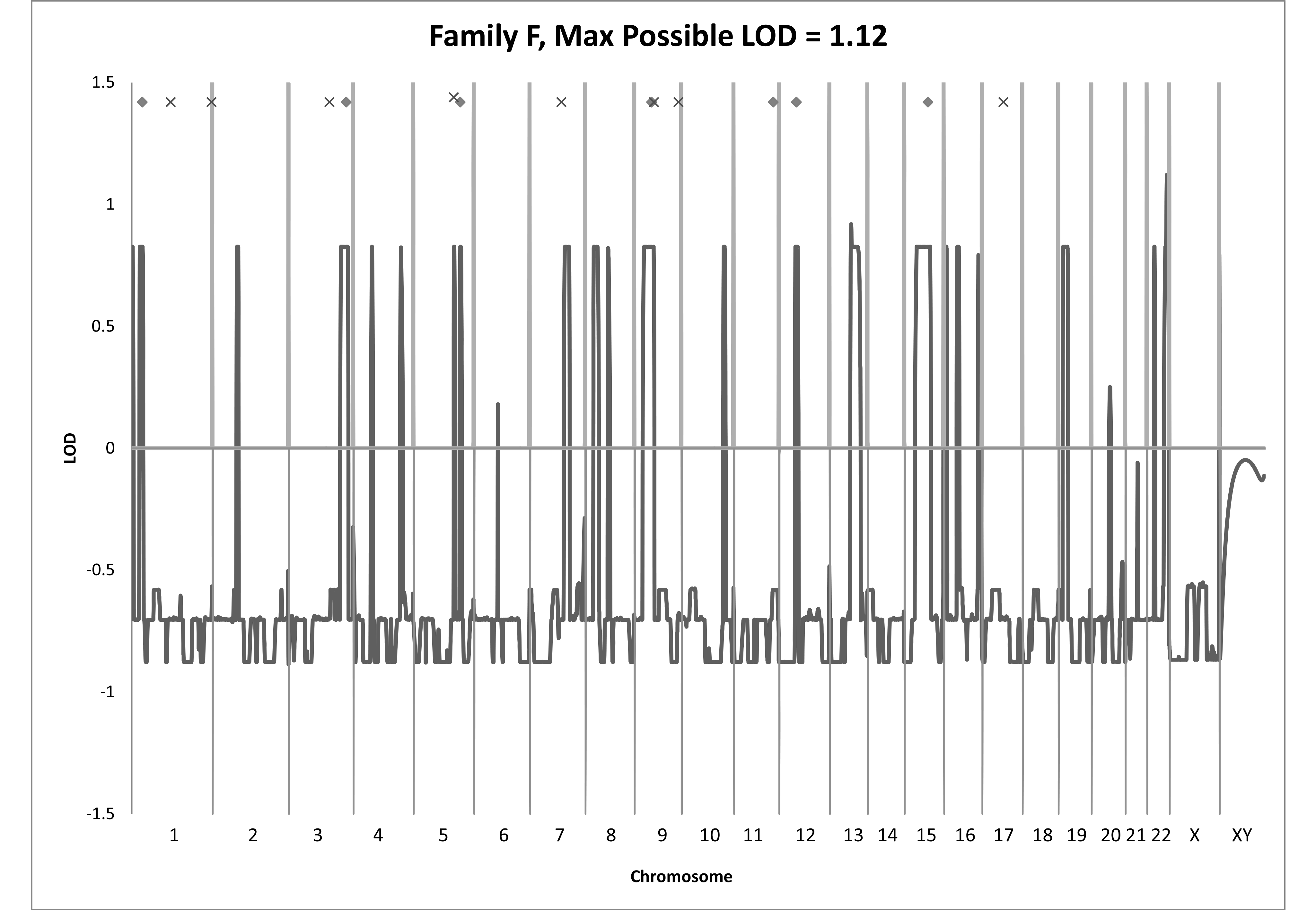

Supplement: S6 Fig — Details of the disease-specific modeling are described in the Methods. Positions of candidate single nucleotide variants and insertion/deletions identified in the whole exome sequencing data are denoted by diamonds and crosses, respectively. (TIF) [file pone.0121104.s006.tif]

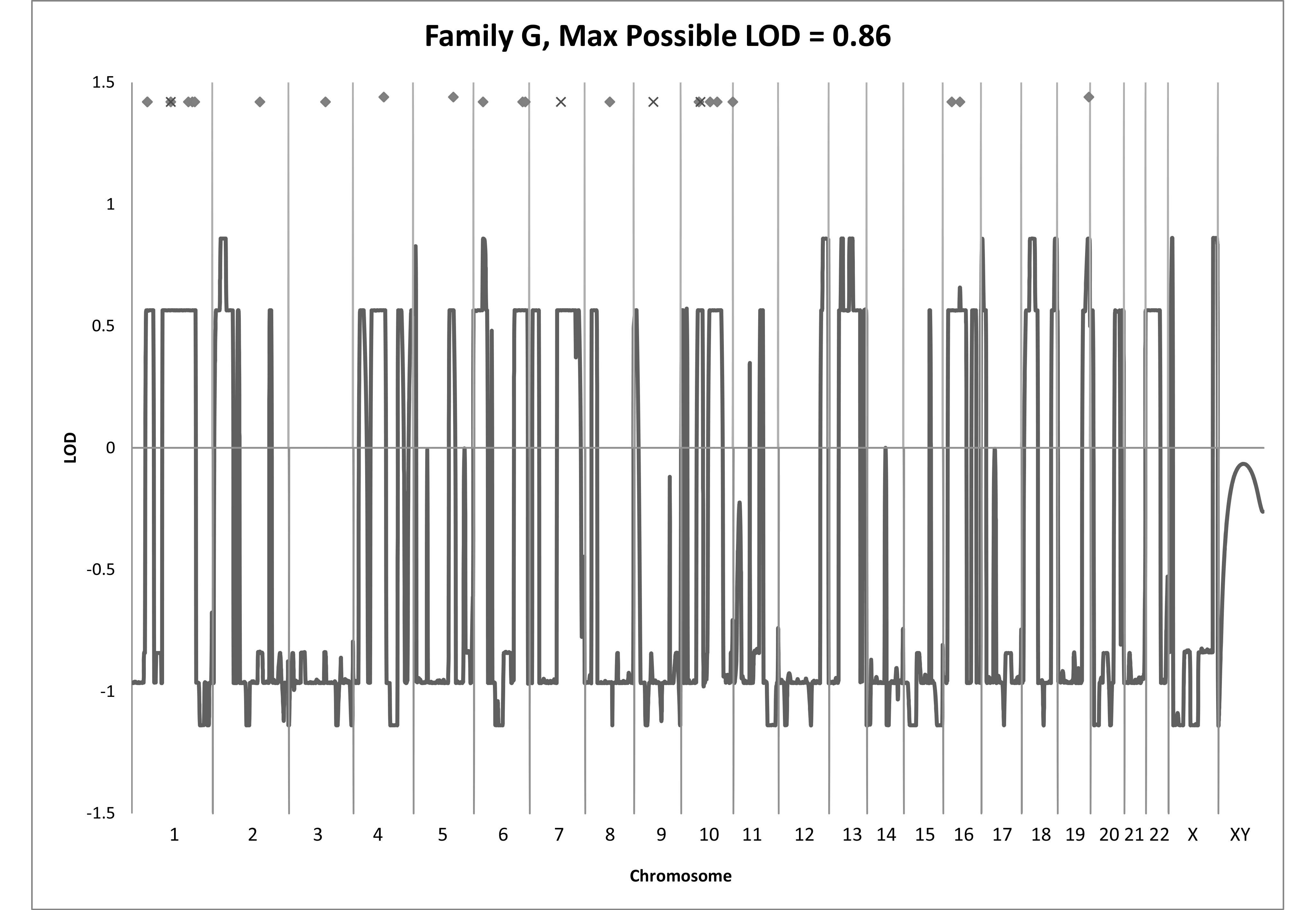

Supplement: S7 Fig — Details of the disease-specific modeling are described in the Methods. Positions of candidate single nucleotide variants and insertion/deletions identified in the whole exome sequencing data are denoted by diamonds and crosses, respectively. (TIF) [file pone.0121104.s007.tif]
